# Galactose-Binding C-Type Lectin Promotes Cellular Aggregation of Coelomocytes in Sea Cucumber

**DOI:** 10.3389/fimmu.2021.783798

**Published:** 2021-12-14

**Authors:** Mizuki Taguchi, Chikaya Tanaka, Shigeyuki Tsutsui, Osamu Nakamura

**Affiliations:** ^1^ School of Marine Biosciences, Kitasato University, Kanagawa, Japan; ^2^ Department of Biology, Tokyo Medical University, Tokyo, Japan

**Keywords:** coelomocytes, cellular aggregation, lectin, cell migration, sea cucumber

## Abstract

Echinoderms have a large coelomic cavity containing coelomocytes. When the coelomic fluid is removed from the cavity, the cells aggregate immediately. We found that a fraction or an extract of the intestine of the sea cucumber, *Apostichopus japonicus*, markedly accelerated cellular movement and aggregation on a glass slide, and this effect was clearly inhibited by galactose. We successfully purified the aggregation-promoting factor, a 16 kDa protein, from the intestine. TOF-MS analysis followed by *de novo* sequencing revealed that the protein is a C-type lectin. RNA-seq data and cDNA cloning demonstrated the factor to be a novel lectin, named AjGBCL, consisting of 158 aa residues in the mature form. Microscopic observation revealed that most of the aggregating cells moved toward aggregates and not to an intestinal fragment, suggesting that AjGBCL is not a chemoattractant but a cellular aggregation-inducing factor that may induce aggregates to release chemoattractant. We report, for the first time, an endogenous molecule that promotes coelomocyte aggregation in echinoderms.

## Introduction

Echinoderms are deuterostomes that possess a large coelomic cavity filled with coelomic fluid (1). This fluid harbors a number of cells called coelomocytes, which play critical roles in defense in these animals. Amoebocytes are among the major types of coelomocytes that exhibit phagocytic activity against bacteria and foreign materials (2). In the case of subjects larger than those that can be phagocytosed, coelomocytes surround and eliminate the target by a process called encapsulation (2–4). Coelomocytes also exert cytotoxic activity against certain types of cells, for example, rabbit erythrocytes and K562 tumor cells (5).

One outstanding phenomenon in which coelomocytes are involved is aggregation; these cells quickly aggregate when they are removed from the coelom (6). This phenomenon is observed in almost all echinoderms. Although the physiological significance of cellular aggregation has not been sufficiently elucidated, it is suggested that this phenomenon is involved in wound sealing (7). When tissue is damaged in vertebrates, the blood coagulation cascade is initiated immediately, resulting in the formation of hemostatic thrombus that seals the wound and prevents the loss of body fluids (8). Moreover, in some invertebrates, such as arthropods, coagulation of hemolymph occurs in response to injury and infection. In horseshoe crabs, granulocytes, recognizing microorganisms, release granules that contain serine protease zymogens—factor C and G. These molecules are autocatalytically activated by lipopolysaccharides (LPS) and β-glucans, respectively, and initiate the coagulation cascades, which result in the conversion of soluble coagulogen to insoluble coagulin gel (9). However, the coagulation of the coelomic fluid does not generally occur in echinoderms (7). It is, therefore, reasonable to assume that cellular aggregation in echinoderms plays a role in the sealing of wounds instead of the coagulation systems (10, 11).

Although aggregation of coelomocytes was first reported by Geddes (12) about 140 years ago, the molecular basis for it has remained almost unclear. To date, only two endogenous molecules related to cellular aggregation in Echinodermata have been reported in sea urchins. Amassin, a 75 kDa olfactomedin protein found in the coelomic fluid of *Strongylocentrotus purpuratus*, strengthens the cell-to-cell crosslinking through disulfide bonds to form larger aggregates of coelomocytes (13). Arylsulfatase in coelomocytes is involved in the cell-to-cell adhesion by crosslinking of sulfated polysaccharides on cell surface in *Lytechinus variegatus* (3). These two proteins, however, neither induce nor accelerate cellular aggregation.

For the formation of aggregates, it is necessary to make free cells move toward and attach to other cells. In a time-lapse observation, we confirmed that crawling coelomocytes, probably amoebocytes, indeed joined to an aggregate on a glass slide (14). This indicates that coelomocytes in the growing aggregates secrete chemotactic agents. Furthermore, such chemoattractants appear to be released from the coelomocytes in response to some stimulation. When removed from the coelomic cavity, coelomocytes immediately begin to aggregate; this indicates that some stimulant, which may be air or unknown molecules released from the injured tissue, induce coelomocytes to secrete the chemotactic agent. Majeske et al. (15) reported that aggregation was augmented by LPS in the sea urchin, *S. purpuratus*, suggesting that molecules related to microorganisms, such as LPS, can accelerate the formation of aggregates, probably by inducing chemotactic molecules. However, no endogenous factor that promotes the release of chemotactic molecules has been identified.

Some species of sea cucumber, including the Japanese sea cucumber, *Apostichopus japonicus*, exhibit an astounding behavior; they eviscerate their own digestive tract when exposed to stress, such as attacks by a natural enemy or changes in the environment (16). When evisceration occurs, most part of the intestine is autotomized and ejected outside with an attached respiratory tree, which brings the animal to a crisis wherein enteric bacteria and external microorganisms can flow into the coelomic cavity. Sea cucumber, therefore, must cover the cut surface of the intestine immediately after evisceration to defend itself against infection by pathogens (17). Assuming that cellular aggregates contribute to wound sealing by covering the sectioned intestinal surfaces, the damage to the intestine should promote cellular aggregation.

In this study, we aimed to verify this hypothesis and identify an aggregation-promoting-factor, which would be contained in the intestine. Here, we report the successful purification and identification of the cellular aggregation-promoting factor from the intestine of *A. japonicus.*

## Material and methods

### Animals

Adult sea cucumbers *Apostichopus japonicus* were purchased from a fishery. They were maintained in a glass aquarium with artificial seawater (SEA LIFE, Marine Tech) at 13°C. After anesthetization with ice, the body wall, tentacles, Polian vesicle, intestine, and respiratory tree were collected and stored at −80°C until use, if they were not used immediately.

### Observation of Cellular Aggregations in Eviscerated Animals

To investigate whether evisceration induces cellular aggregation and wound sealing, we autotransplanted fluorescent-labeled coelomocytes to the animals after evisceration. First, coelomic fluid was obtained using a syringe with 23-gauge needle (Terumo), mixed with an equal volume of Calcein-AM (0.5 mg/ml, Dojindo), and stirred for 10 min. The cells were washed three times with a mixture of 2.4% NaCl, 0.6% KCl, and 0.03 M PBS (3× PBS, pH 7.2) and resuspended in cell-free coelomic fluid (5×10^6^ cell/ml). Next, sterilized distilled water (SDW) (5% of the body weight) was injected into the same individuals to induce evisceration of the digestive tract. After evisceration, 3 ml of the fluorescently labeled coelomocytes was autografted to the coelomic cavity and the animals were kept in an aquarium for 3 h. After anesthetization with ice, the specimen was cut on the ventral side, and a section of the intestinal tissue was observed under a fluorescence microscope (Axioskop2, ZEISS, objective lens ×4) at 25°C. Images were acquired using a digital camera (Axiocam HRc, Zeiss) and the software provided with it (Axiovision, Zeiss), and the contrast was enhanced using the Image J software.

### Microscopic Observation of Cellular Aggregation

To determine the effects of the intestinal tissue on cellular aggregation *in vitro*, the freshly obtained intestine was cut into 5 mm square sections and fixed on a glass slide with a drop of 1% agarose solution, which gelled immediately. Fresh coelomic fluid was collected from an animal using a syringe with 23-gauge needle (Terumo). Fifty microliters of the coelomic fluid was dropped onto the intestinal section on a glass slide, covered with a cover slip, and incubated for 30 min. Concomitantly, the same volume of coelomic fluid was dropped onto a glass slide without an intestinal section. The aggregates that were formed were observed under a light microscope (DP26, Olympus), and their areas were measured using the region-of-interest analysis function of the cellSens standard program (version 1.6).

### Effect of Tissue Extracts on Cellular Aggregation

Each tissue, including the body wall, tentacles, intestine, respiratory tree, and Polian vesicle, was homogenized with an equal volume of 3× PBS. After centrifugation at 8,200 × *g* for 30 min, the supernatant was collected as the tissue extract. Forty microliters of the coelomic fluid and 10 μl of the tissue extract or 3× PBS (as a control) were mixed and placed on a glass slide. Observation and measurement of the area of the aggregates were performed as mentioned above.

### Ultrafiltration or Heat Treatment of the Intestinal Extract

Five microliters of the intestinal extract was centrifuged at 8,200 × *g* for 15 min using an ultrafiltration device (Vivaspin 5k, Sartorius). After centrifugation, the filtrates and concentrates were collected separately. To examine the thermostability of the aggregation-promoting factor, the intestinal extract was heated at 95°C for 5 min. These preparations were mixed with the coelomic fluid at a ratio of 1:9 and cellular aggregation was observed as described above.

### Purification of the Aggregation-Promoting Factor

First, crude protein was extracted from the intestine according to the procedure described by Ishimoda-Takagi and Ozaki (18) with slight modifications, because we suspected the aggregation-promoting factor to be tropomyosin. The intestine was minced and mixed with an equal volume of SDW and incubated for 30 min. The mixture was filtered through a nylon mesh with a pore size of 100 μm, and the residue was serially washed with 100% ethanol, 50% ethanol, and 97% ethanol. The residue was dried at 20°C and dissolved in 1 M KCl (7 ml/g). The pH of the solution was adjusted to 7 with NaOH and the solution was incubated at 20°C for 12 h. After centrifugation (8,200 × *g* for 30 min), the supernatant was collected and its pH was adjusted to 4.0 with HCl. After incubation for 1 h and centrifugation (8,200 × *g* for 30 min), the supernatant was collected and applied to an anion-exchange column of POROS HQ/20 (Applied Biosystems) equilibrated with 50 mM Tris-HCl (pH 8.0) using the BioLogic DuoFlow system (Bio Rad). The bound proteins were eluted with NaCl applied in a gradient from 0 to 0.5 M in 50 mM Tris-HCl. Each eluted fraction was added to the coelomic fluid and the aggregation-promoting activity was examined by microscopic observation. The fraction with the highest activity was subjected to gel-filtration chromatography using a Superdex 200 column (GE Healthcare) equilibrated with 50 mM Tris/HCl containing 150 mM NaCl (pH 7.5). The aggregation-promoting activity of each fraction was examined in the same manner and the fraction with the highest activity was subjected to SDS-PAGE.

### Inhibition of the Aggregation-Promoting Activity by Carbohydrates

To verify our speculation that the aggregation-promoting factor could be a lectin, we examined whether the activity was inhibited by carbohydrates. The intestinal extract was mixed with an equal volume of 0.1 M solution of D (+)-glucose, D (+)-mannose, D (+)-galactose, D (+)-xylose, L (−)-fucose, L (+)-rhamnose, *N-*acetyl-D-glucosamine, *N-*acetyl-D-galactosamine, D (+)-maltose, D (+)-lactose, or D (+)-sucrose solution (in PBS), and incubated overnight. Thereafter, 10 μl of the mixture was added to 40 μl of the coelomic fluid and mounted on a glass slide, covered with a cover slip, and incubated for 30 min in a moist chamber. Observation of the aggregates and measurement of their area were done as mentioned above.

### Affinity Chromatography

To purify the galactose-binding protein from the intestinal extract, 1 ml of galactose-agarose (Thermo Scientific) and 4 ml of the extract were mixed and gently stirred at 4°C overnight. The non-adsorbed fraction was removed and the galactose-agarose was washed with 3× PBS. The adsorbed protein was eluted with 4 ml of 0.2 M galactose-PBS.

### *De Novo* Sequencing

A single 16 kDa band detected on a SDS-polyacrylamide gel after gel-filtration chromatography was subjected to liquid chromatography coupled to time-of-flight high resolution mass spectrometry (LC/Q-TOF/MS; TripleTOF 5600; SCIEX), followed by *de novo* sequencing. The band on the gel was excised and heated in 100 μl of 50 mM NH_4_HCO_3_ and 1 μl of 1 M dithiothreitol at 80°C for 10 min for reductive alkylation of the protein. Thereafter, 2 μl of 1 M iodoacetamide was added and incubated at 37°C for 15 min. After removing the liquid, 100 μl of 100% methanol and 100 μl of 10% acetic acid were added to destain the gels. The gels were contracted by shaking in 100% acetonitrile for 5 min. After the gels were dried, 20 μl of trypsin solution (0.5 mg/ml) in 50 mM NH_4_HCO_3_ was added and incubated overnight at 37°C. The digested proteins were analyzed by LC/Q-TOF/MS with the Analyst TF 1.7 Software (SCIEX). Chromatographic separation was carried out with a Cadenza CD-C18 column (150 × 2 mm, Imtakt, Japan). Elution buffers were 0.5% formic acid in SDW (solvent A) and acetonitrile (solvent B). Raw data were analyzed using the PeakView Software (SCIEX) and DeNovoGUI (Compomics). The single band detected on SDS-polyacrylamide gel after galactose-affinity chromatography was also analyzed in the same manner.

### RNA-Seq Analysis

Total RNA was isolated from the intestine and coelomocytes of *A. japonicus* using ISOGEN (Nippon Gene). The quality and quantity of RNA were assessed with a Nanodrop 2000c spectrophotometer (Thermo Scientific) and 2100 Bioanalyzer using the RNA 6000 Nano kit (Agilent Technologies). A cDNA library was prepared from 1 µg of total RNA, a mixture of equal parts of total RNA from the intestine and CC, using NEBNext Ultra Directional RNA Library Prep Kit for Illumina (New England BioLabs), NEBnext Poly (A) mRNA Magnetic Isolation Module (New England BioLabs), and NEBNext Multiplex Oligos for Illumina (Dual Index Primers Set 1, New England BioLabs) according to the manufacturer’s protocol. The protocol was amended to lower the fragmentation time from 15 min to 10 min to generate larger RNA fragments. The quantity of cDNA library was assessed on a KAPA Library Quantification Kit for Illumina platforms (KapaBiosystems). Sequencing was performed with Miseq (Illumina) using 301 bp paired-end reads with a Miseq reagent kit v3 (600 cycles). The sequencing reads were trimmed on the 3′-end using the statistical software R version 3.4.0 (19) with ShortRead package [version 1.34.0; (20)] and Biostrings package [version 2.44.1; (21)]. Next, trimmed reads were merged using BBmerge (https://sourceforge.net/projects/bbmap/; parameters: verystrict=t). Thereafter, low-quality reads were removed using the FASTX-toolkit (http://hannonlab.cshl.edu/fastx_toolkit/; parameters: -q 30, -p 80). Transcriptome assembly was performed using Trinity version 2.5.1 [ (22); parameters: -SS_lib_type R, min_contig_length 200).

### cDNA Cloning and Identification of the Aggregation-Promoting Factor

Total RNA was isolated as mentioned above and cDNA was prepared using the SMART™ RACE cDNA amplification kit (Clontech). Sequences similar to those of the peptides obtained by *de novo* sequencing were searched by BLAST. The hits in the BLAST search were used as queries to search for homologs in the RNA-seq data for the intestine and coelomocytes. Because one sequence that was highly identical to multiple peptide sequences obtained by *de novo* sequencing was found in the RNA-seq data, cDNA sequencing was performed to confirm the sequence. A pair of primers, Aj-GBP3-1 (5′-TTC GGT CGA CTT TAC AAT ACA CCA ACA G-3′) and Aj-GBP5-1 (5′-TAC ATC GGC CCA TTT ACC ACG CGA ATG C-3′), was designed based on the sequence. PCR was performed using Taq polymerase (Ex Taq, Takara) under the following conditions: 95°C for 3 min; 30 cycles of 95°C for 30 s, 64°C for 30 s, 72°C for 30 s; and 72°C for 7 min. The amplicon was ligated into the pGEM-T Easy vector (Promega) and introduced into *Escherichia coli*-DH5α competent cells (HIT, RBC Bioscience). DNA sequencing was performed using a Big-Dye Terminator Cycle sequencing kit ver.3.1 (Applied Biosystems) and DNA sequencer (3100-Avant, Applied Biosystems). The 3′-RACE PCR was performed using cDNA extracted from the intestine, Ex Taq, a forward primer, Aj-GBP3-1, and a reverse adapter primer, NUP (5′-AAG CAG TGG TAT CAA CGC AGA GT-3′, Clontech). The conditions for 3′-RACE PCR were the same as described above. A phylogenetic analysis was performed to identify the homologous molecules. Sequences similar to that of the aggregation-promoting factor of *A. japonicus* were searched using local BLAST of the 807 echinoderm C-type lectin sequences registered in the NCBI database. Of these, 191 sequences had an E-value above 10^-5^ and were selected for subsequent analyses. Phylogenetic trees were constructed using the maximum likelihood method with 100 bootstrap replicates based on 191 sequences using MEGA X software.

### Gene Expression Analysis

Total RNA was extracted from the body wall, tentacles, Polian vesicle, intestine, respiratory tree, and coelomocytes, and reverse transcribed as described above. The expression of *AjGBCL* in these tissues was examined using a pair of specific primers, Aj-GBP3-2 (5′-CCA TAG CAT CGG GCT TGC ATT CGC GTG G-3′) and Aj-GBP5-2 (5′-TAC ACC TGT GGA TCC CAA CTG TTG GTG-3′), and Ex Taq. The PCR conditions were as follows: 95°C for 3 min; 30 cycles of 95°C for 30 s, 64°C for 30 s, and 72°C for 30 s. The expression of actin gene was used as an internal control, using a pair of primers, Actin-F (5′-TCC TTC ACC ACC ACA GCC GA-3′) and Actin-R (5′-ACC GGC GGA CTC CAT TCC AA-3′), which were designed based on the sequence of the *A. japonicus* actin gene (BAH79732).

### Characterization of Aggregated Cells

Ten milliliters of the coelomic fluid was collected and divided into two 50 ml centrifuge tubes. Five hundred microliters of the intestinal extract was added into one tube, and the same volume of 3× PBS was added to the other as a control. The mixture was allowed to stand for 30 min and passed through a nylon mesh with a pore size of 100 μm to separate the aggregates. After washing three times with 3× PBS, the mesh was turned over and the aggregates were collected and incubated with 200 mM EDTA for 2 h to dissociate the aggregated cells. The dissociated cells were counted using a Burker Turk hemocytometer, and 5.0 × 10^5^ cells were centrifuged onto a glass slide. After staining with May–Grunwald/Giemsa stain, 300 randomly selected cells were classified as described by us in a previous study (15).

### Statistical Analysis

The statistical significance of differences in the total area of cellular aggregates, with or without the piece of intestine, was examined using the paired *t*-test with the PRISM software (Graphpad). In the aggregation experiment using several tissue extracts, the significance of the difference was analyzed by repeated measurement of ANOVA followed by Tukey’s multiple comparison test. Differences in the cellular composition of the aggregates, with or without the intestinal extract, were analyzed by paired *t*-test. Differences were considered significant at *p* < 0.05.

## Result

### Autologous Transplantation of the Coelomocytes to the Eviscerated Animal

**Figure 1:**
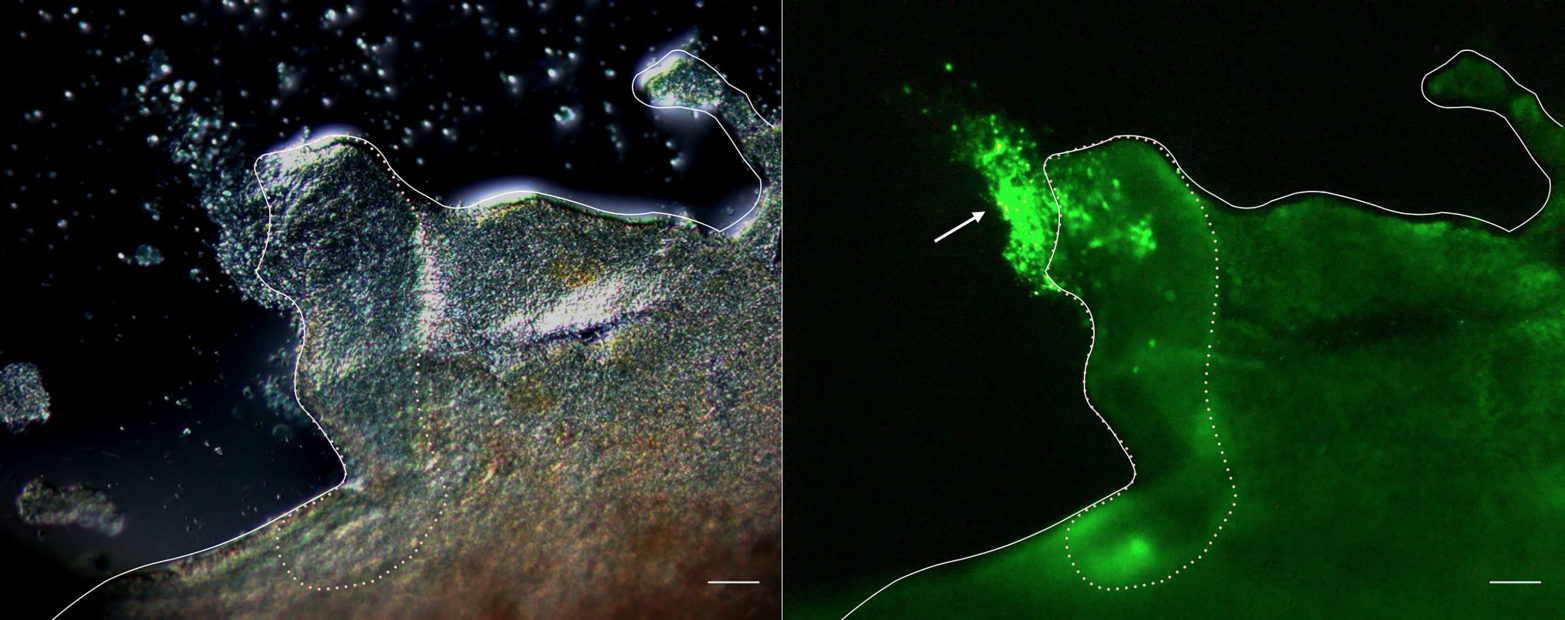
Fluorescence microscopy of the cleavage site of the intestine. Images taken 3 h after autotransplantation of labeled coelomocytes to the eviscerated animal. Left panel: Differential interference contrast. Right panel: Image showing the fluorescence. Full line: Serosa. Dotted line: Cleavage site. →: cellular aggregates. Scale bars = 200 μm.

To verify the role of cellular aggregation upon tissue damage, the evisceration of *A. japonicus* was artificially induced, and fluorescently labeled coelomocytes were autografted into the coelom of the animals. As shown in **Figure 1**, fluorescence signals were detected on the sectioned surface of the digestive tract at 3 h after this autologous transplantation. This indicates that the aggregates of coelomocytes cover the surface of the digestive tract and seal it after evisceration.

### The Aggregation-Promoting Activity of the Intestinal Tissue

**Figure 2:**
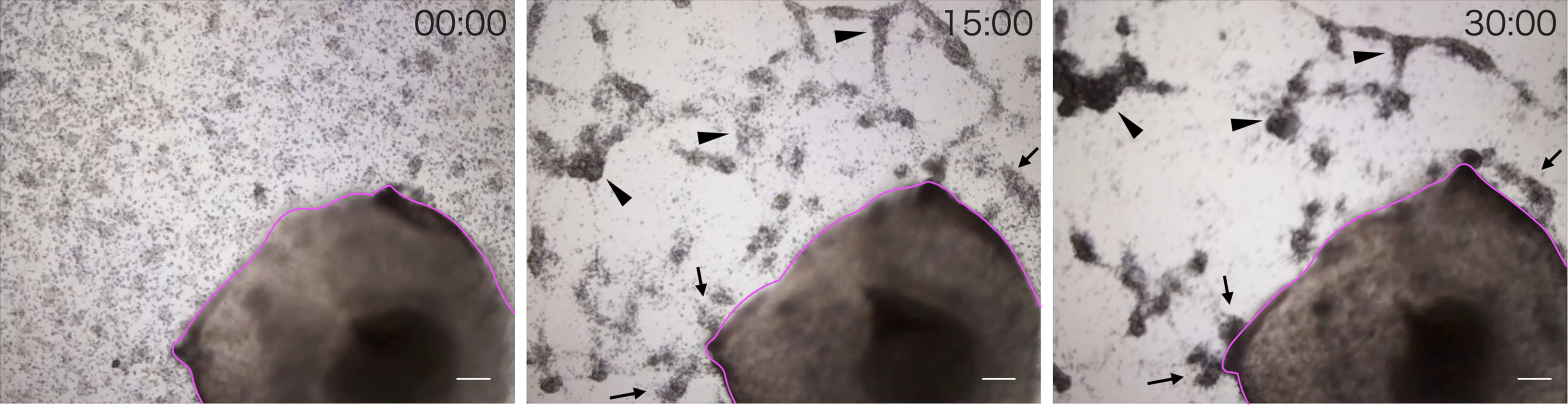
﻿Time-lapse observation of the movement of cells toward a piece of intestine. Images were acquired every 15 min. Magenta line: A piece of intestine. Arrow: ﻿Cells moved toward the growing aggregates. Arrowhead: Cells moved toward the piece of intestine. Scale bars = 200 µm.

**Figure 3:**
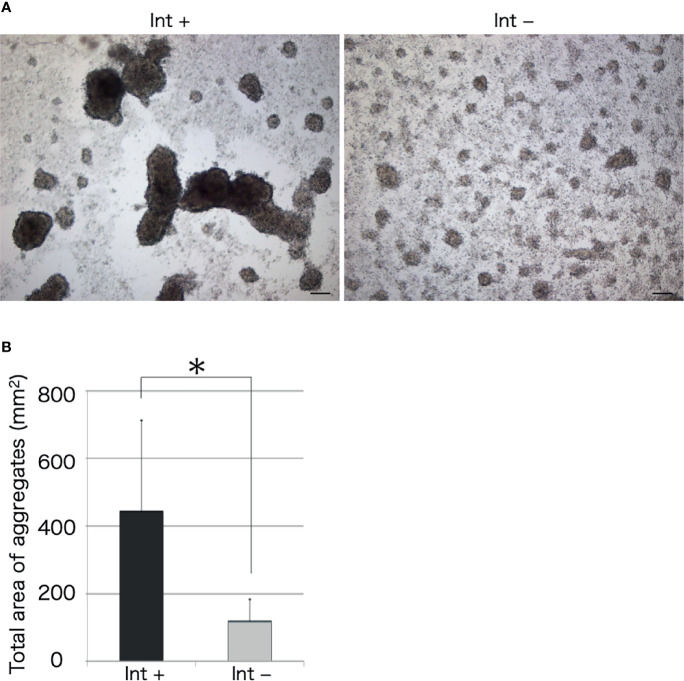
Microscopic observation of cellular aggregates with (Int+) or without (Int-) an intestinal piece on a glass slide. **(A)** Aggregates formed in the presence and absence of the intestine at 30 min. Scale bars = 100 μm. **(B)** Comparison of the total area of cell aggregates. *P < 0.05 (mean ± SD, n = 5).

We observed the aggregation of coelomocytes microscopically in the presence of a piece of intestinal tissue. Time-lapse observation revealed that the coelomocytes moved and aggregated more quickly and formed larger aggregates in the presence of the tissue (**Supplementary Video 1**). Most of these cells moved toward the growing aggregates, but rarely toward the piece of intestine (**Figure 2**). However, a few aggregates in the vicinity of the tissue eventually got attached to it. After 30 min, the total area of the aggregates formed under this condition was about 4–5-fold larger than that in the control (**Figure 3**). This suggests that the intestinal tissue contains substances that promote cellular aggregation of the coelomocytes.

**Figure 4:**
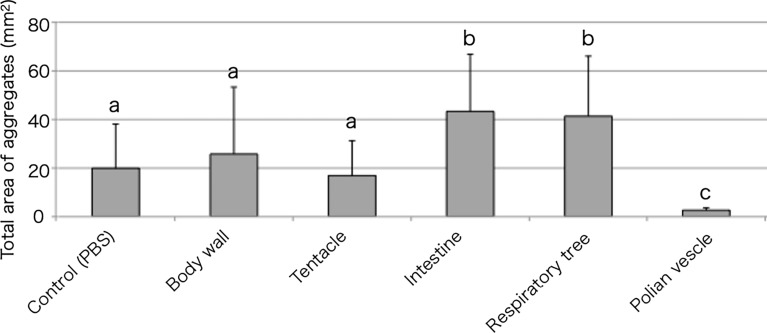
Aggregation-promoting activity of the tissue extracts. Total area of the aggregates, 30 min after the addition of the extracts to the coelomic fluid. *P* < 0.05 (mean ± SD, n = 5). Different letters show significant differences.

Tissue extracts of the body wall, tentacle, intestine, respiratory tree, or Polian vesicle were also examined for their effect on aggregation (**Figure 4**). The intestinal extract markedly accelerated the cellular aggregation. Other than the intestinal extract, only the respiratory tree extract showed this effect. Interestingly, the extract of the Polian vesicle inhibited the cellular aggregation.

**Figure 5:**
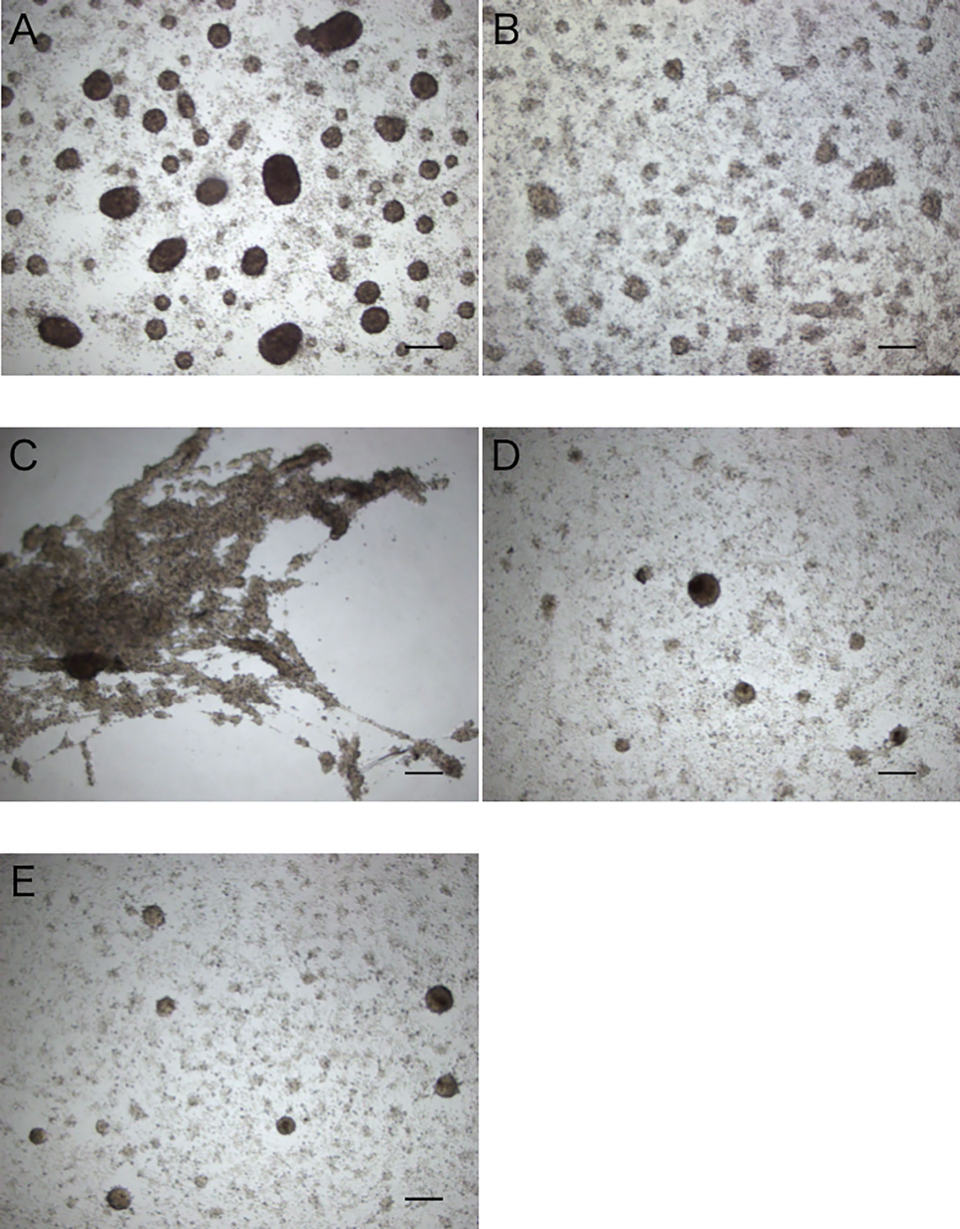
Aggregation-promoting activity of the treated extracts of the intestine. **(A)** untreated extract. **(B)** heat-treated extract. **(C, D)** >5 kDa and <5 kDa fractions of the extract separated by ultrafiltration. **(E)** control (coelomic fluid only). Scale bars = 200 μm.

When the intestinal extract was subjected to heat treatment, the total area of the aggregates formed was decreased to nearly the same as that in the control, indicating that the aggregation-promoting factor is heat unstable (**Figures 5A, B, E**). After ultrafiltration of the extract through a 5 kDa membrane, a strong aggregation-promoting effect of the concentrated extract was observed, whereas no effect was observed for the filtrate (**Figures 5C–E**). These results suggested that the factor is a protein.

### Purification of the Aggregation-Promoting Factor

**Figure 6:**
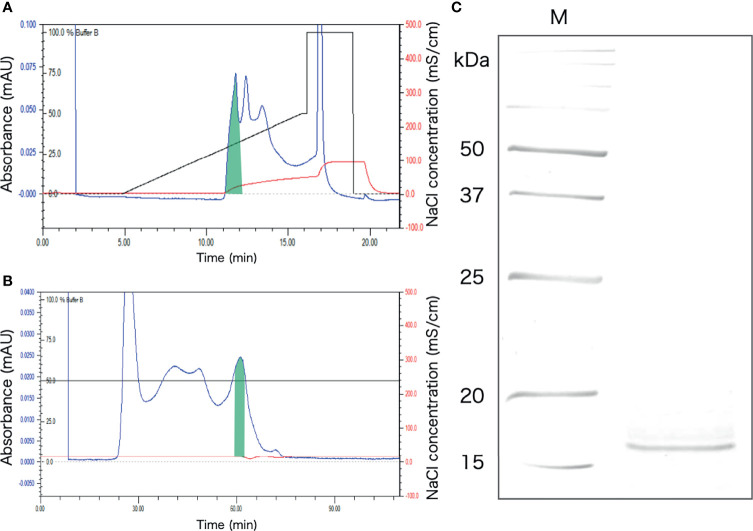
Purification of the aggregation-promoting factor from the crude protein fraction of the intestinal extract. **(A)** Chromatogram after anion-exchange chromatography on a POROS HQ/20 column. The fraction with the activity is shaded. **(B)** Chromatogram after gel-filtration chromatography on a Superdex 200 column. The shaded fraction in **Figure 4A** was applied. A fraction with the activity is shaded. **(C)** SDS-PAGE of the shaded fraction in **Figure 4B**. A 15% polyacrylamide gel was used and was visualized by staining with coomassie brilliant blue. M, marker.

The protein extracted from the intestinal extract was subjected to anion-exchange chromatography, followed by gel filtration chromatography (**Figures 6A, B**). After anion-exchange chromatography, the aggregation-promoting activity was detected in the fraction indicated in **Figure 6A**. This fraction was subjected to gel-filtration chromatography, and one of the eluted fractions exhibited the activity (**Figure 6B**). When the fraction was subjected to SDS-PAGE, only a single 16 kDa band was observed (**Figure 6C**).

### Inhibition of the Aggregation-Promoting Activity by Sugars

**Figure 7:**
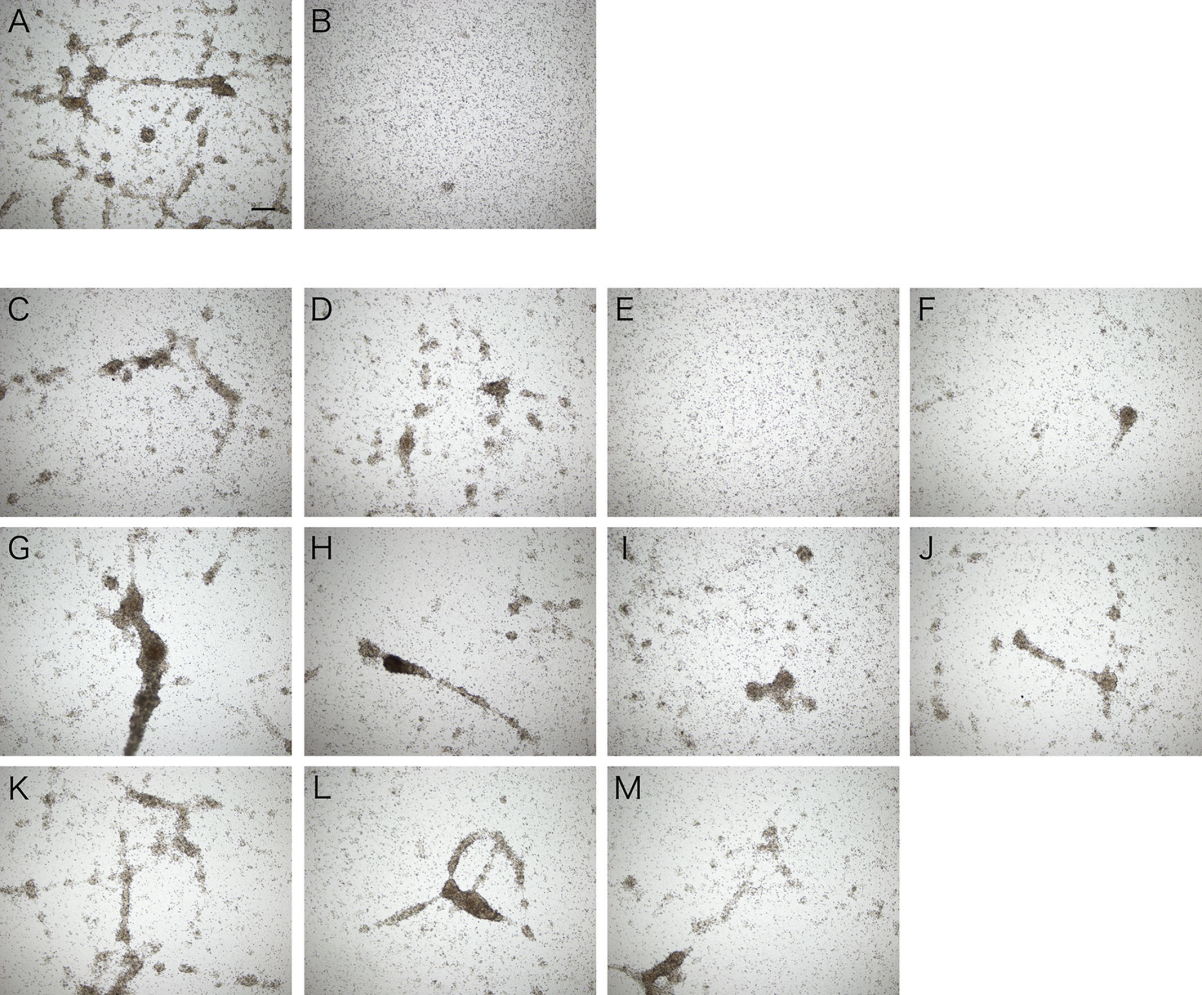
Inhibitory effect of galactose on cellular aggregation. Microscopic observation. Coelomic fluid was supplemented with **(A)** the intestinal extract, **(B)** PBS, or **(C–M)** the intestinal extract premixed with 0.1 M sugar solution (in PBS). **(C)** D (+)-glucose. **(D)** D (+)-mannose. **(E)** D (+)-galactose. **(F)** D (+)-xylose. **(G)** L (−)-fucose. **(H)** L (+)-rhamnose. **(I)** *N-*acetyl-D-glucosamine. **(J)** *N-*acetyl-D-galactosamine. **(K)** D (+)-maltose. **(L)** D (+)-lactose. **(M)** D (+)-sucrose. Scale bars = 200 μm.

Assuming that the aggregation-promoting factor is a lectin, we examined the inhibitory effect of sugars on the aggregation-promoting activity of the intestinal extract. The activity was markedly inhibited by galactose but not by other sugars (**Figure 7**). This suggests that the aggregation-promoting factor is a galactose-binding protein.

**Figure 8:**
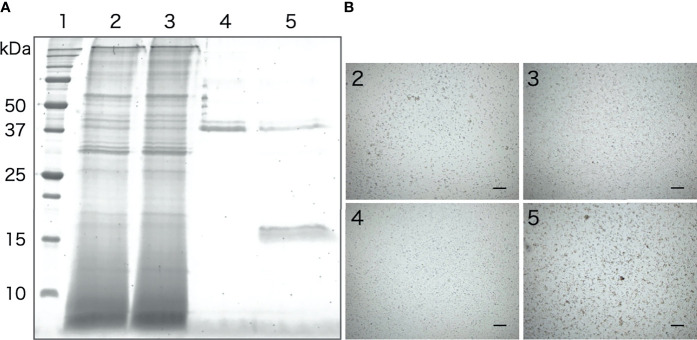
Purification of the aggregation-promoting factor from the intestinal extract by galactose-affinity chromatography. **(A)** SDS-PAGE. Lanes: 1, molecular mass maker; 2, Intestinal extract; 3, non-adsorbed; 4, rinse-off; 5, galactose-binding protein. **(B)** Aggregation-promoting effects of each fraction. The numbers on the panels correspond to the number of the lanes in **Figure 7A** .Microscopic observation. Scale bars = 200 μm.

The galactose-binding protein was purified from the intestinal extract by affinity chromatography using galactose-agarose. As shown in **Figure 8A**, we detected a single band of about 16 kDa, similar to that of the aggregation-promoting factor purified by anion-exchange and gel-filtration chromatography, in the fraction eluted with galactose. When the eluted fraction was supplemented to the coelomic fluid, cellular aggregation was accelerated (**Figure 8B**), indicating that the protein was indeed the aggregation-promoting factor.

### Identification of the Aggregation-Promoting Factor

**Figure 9:**
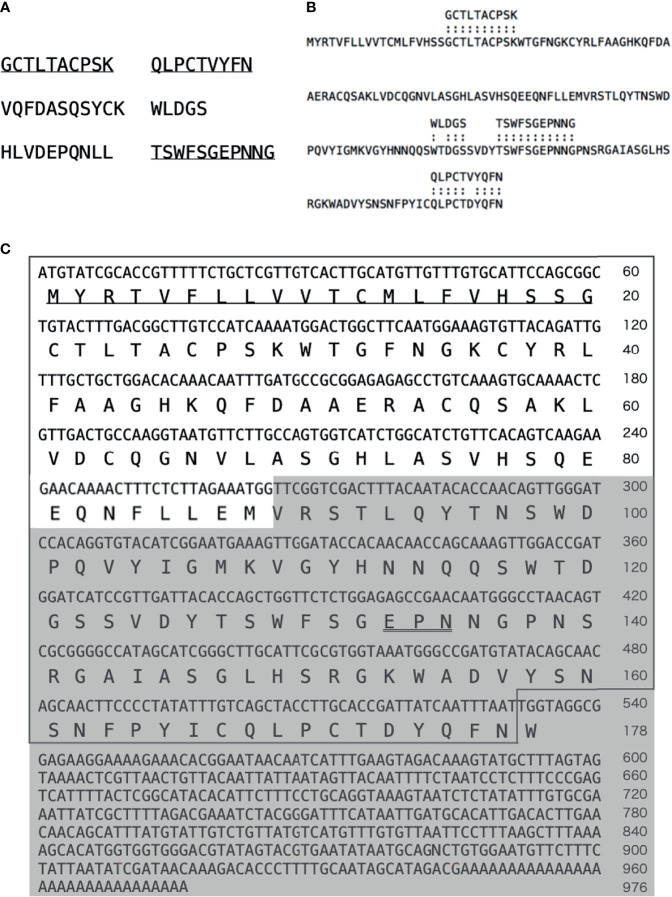
Identification of the aggregation-promoting factor. **(A)** Peptide sequences obtained by *de novo* sequencing of the 16 kDa protein purified by anion-exchange and gel-filtration chromatography (**Figure 5C**). The sequences obtained by *de novo* sequencing of the protein purified by galactose-affinity chromatography (**Figure 7A**), as well, are underlined. **(B)** A sequence found in RNA-seq data that is highly identical to the peptide sequences. **(C)** cDNA sequence and deduced amino acid sequence of AjGBCL. The sequence obtained by RNA-seq is boxed. The sequence of partial and 3′-RACE amplified cDNA confirmed by sequencing in shaded. An EPN motif is double underlined.

*De novo* sequencing of the 16 kDa protein purified by gel-filtration chromatography provided six peptide sequences, and that of the protein obtained by the galactose-affinity chromatography gave three peptide sequences, all of which were also present in the former (**Figure 9A**). Protein sequences were subjected to BLAST search, which revealed that these peptides have similarities to C-type lectins of *A. japonicus* registered in GenBank (ABC87994 and PIK42223). Sequences similar to these lectins were then searched in the RNA-seq data. We found one sequence with high identity to four peptide sequences obtained by *de novo* sequencing, which included the three sequences mentioned above (**Figure 9B**). Two sequences that do not match with AjGBCL (**Figure 9A**) did not hit any sequences deposited in databases. We then performed cDNA cloning to determine the sequence, and identified that the cDNA of the aggregation-promoting factor includes a 534 bp open reading frame, encoding a protein with 178 aa residues, and having a 20 aa predicted N-terminal signal peptide (**Figure 9C**). BLAST search of this sequence did not give any identical sequence; however, similarity with C-type lectins of echinoderms was observed. These data indicate that the aggregation-promoting factor is a galactose-binding C-type lectin that has not been reported. Hereafter, the aggregation-promoting factor from *A. japonicus* will be referred to as “AjGBCL”, a short form for “*A. japonicus* galactose-binding C-type lectin”. The original contributions presented in the study are publicly available. This data can be found here: (https://www.ncbi.nlm.nih.gov/nuccore/LC552681.1), Accession No. LC552681.

To determine whether homologues of AjGBCL are present in other echinoderm animals, we performed a homology search and phylogenetic analysis. The closest sequence was a C-type lectin of *A. japonicus*, whose amino acid sequence was 81% similar to that of AjGBCL (**Supplementary Figure 1**). In other species *(Strongylocentrotus purpuratus)*, a sequence designated as the bone morphogenetic protein-1-like was located in the closest branch; however, the bootstrap value of this node was quite low. This protein showed only 35% homology with AjGBCL. Bootstrap values at some nodes in this tree were quite low, probably due to their moderate resemblance to each other. However, no other sequence with high homology with AjGBCL was found.

### Expression Analysis of AjGBCL in Tissues

**Figure 10:**
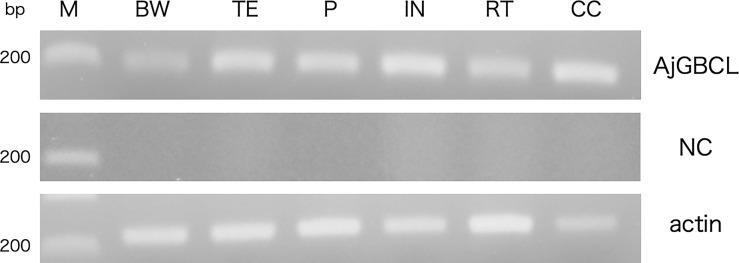
Reverse transcription-PCR analysis of AjGBCL expression in different tissues. β-actin was used as a loading control. M, marker; BW, body wall; TE, tentacles; PV, Polian vesicle; IN, intestine; RT, respiratory tree; CC, coelomocytes; NC, negative control (without the reverse transcription of RNA).

Reverse transcription-PCR showed that *AjGBCL* was expressed in all the examined tissues, namely Polian vesicle, body wall, tentacles, intestine, respiratory tree, and coelomocytes (**Figure 10**). We also confirmed that all the sequences of the amplicons were identical to that of *AjGBCL.*

### Cellular Composition of the Aggregates Formed in the Presence of the Intestinal Tissue

**Figure 11:**
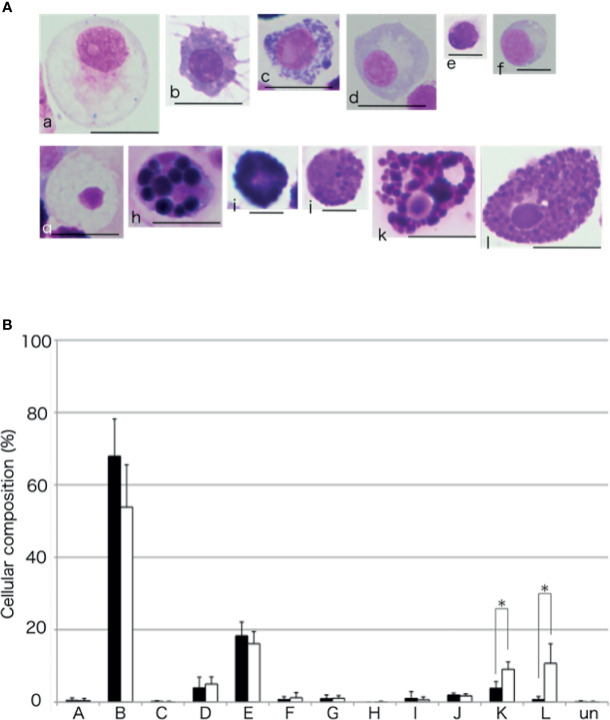
Cellular composition of the aggregates. **(A)** ﻿The coelomocytes of *A. japonicus* classified based on MG staining. a to l indicate Type-A to Type-L, respectively. ﻿ Un, unidentified. Scale bars are 5 (e and f) or 10 mm (a, b, c, d, g, h, i, j, k, and l). **(B)** Comparison of cellular composition of aggregates formed in the presence or absence of the intestinal extract. opened bar; absence, closed bar; intestinal extract. *P < 0.05 (mean ± SD, n = 5).

To compare the cellular composition of the aggregates formed with or without the intestinal extract, the aggregates were dissociated and the cells were classified (**Figure 11**), based on the classification presented in our previous study (15). Amoebocyte was the most abundant cell type without the supplementation of the intestinal extract. However, the proportion of amoebocytes to the total number of cells was lower and those of some other cell types were higher (up to 15%) in the presence of the intestinal extract. In particular, the proportions of the two types of basophilic granulocytes, defined as type K and L in our previous study, were significantly higher than in the case of control (addition of 3× PBS).

## Discussion

The phenomenon of aggregation of the coelomocytes of echinoderms has been known since the 19th century; however, the molecular basis for it has remained almost unclear. In the present study, we identified, for the first time, an endogenous molecule, AjGBCL, that promotes aggregation of the coelomocytes of echinoderms. AjGBCL is a C-type lectin composed of 158 aa residues in its mature form. It possesses a typical carbohydrate-recognition domain of the C-type lectins, which includes an EPN motif. In vertebrate C-type lectins, the EPN motif is considered to be related to a higher specificity for mannose (23). However, several C-type lectins to which this rule does not apply are also known (24, 25).

AjGBCL appeared to stimulate coelomocytes to aggregate through lectin–carbohydrate interaction, because the promotion of aggregation by AjGBCL was markedly inhibited by galactose. This indicates that AjGBCL binds to the receptor molecules possessing β-galactoside chains on the coelomocytes, which in turn transduces the intracellular signals.

It should be noted that the acceleration of cellular aggregation by AjGBCL is not the result of agglutination of the cells by the lectin. We observed the induction of cellular aggregation by AjGBCL on glass slides—to form aggregates, the cells must crawl to join together. Actually, no aggregation occurred when the intestinal extract was added to PFA-fixed coelomocytes on a glass slide (data not shown), indicating that AjGBCL stimulates the coelomocytes to move and form aggregates.

How AjGBCL promotes cellular aggregation remains unclear. There are two possibilities: AjGBCL acts as a chemoattractant by itself or it induces certain cells to secrete chemoattractant materials. To date, only a few lectins that cause chemotaxis have been reported. To the best of our knowledge, in mammals, two galectins, namely galectin-3 and galectin-9, and macrophage-derived neutrophil chemotactic factor have been reported to attract leukocytes directly. Galectin-3 acts as a chemoattractant for human monocytes and macrophages, and galectin-9 induces the migration of human eosinophils (26, 27). Macrophage-derived neutrophil chemotactic factor can attract neutrophils (28). However, to our knowledge, no C-type lectin has been reported to exhibit chemoattractant activity. In time-lapse microscopic observation of cellular aggregation in the presence of a piece of the intestine, most of the coelomocytes migrated toward the growing aggregates, but not toward the intestine. This suggests that AjGBCL does not act as a chemoattractant; instead, it has an effect on particular cells to release the chemoattractant molecules.

Cell migration activating factors of invertebrates are poorly understood. DeVries et al. (29) reported that homologs of vertebrate chemokines and chemokine receptors have not been found in the genomes of invertebrates, including those of Urochordata and Arthropoda. Recently, Furukawa et al. (30) demonstrated that two molecules, *Ap*MIF1 and *Ap*MIF2, which are homologs of mammalian macrophage migration inhibitory factor (MIF), regulate the migration of mesenchymal cells in the larvae of starfish, *Asterina pectinifera*. *Ap*MIF1 and *Ap*MIF2 function to suppress or promote the migration of mesenchymal cells in response to foreign substances injected into the body of larvae, respectively. In 2019, Lv et al. (31) reported the cloning of a gene of an MIF homolog from *A. japonicus* (*Aj*MIF). They showed that bacterial stimulation induced the upregulation of *Aj*MIF in the coelomocytes. They also showed that recombinant *Aj*MIF promotes inflammatory response. The study did not, however, report the chemoattractant activity of the protein. Thus, it is of our interest to examine whether this protein is involved in the aggregation of coelomocytes.

Overall, we hypothesize the following: AjGBCL released from wounded intestine binds to glycoside chains of the receptor on the surface of a certain type of coelomocytes. By intracellular signal transduction, the coelomocytes are activated and release chemotactic molecules, which might be an MIF-homolog. This activates other cells in turn and then induces their migration to the growing aggregates.

In addition, we considered whether AjGBCL-like molecules also play a role in other echinoderm species. As described in the results, we could not find highly-homologous sequences. However, echinoderm animals possess an array of C-type lectin genes; for example, more than 150 C-type lectin sequences of *A. japonicus* have been deposited in various databases. The functions of the proteins encoded by these genes are nearly unknown. In addition, genomic information on echinoderms is limited. Therefore, it is possible that AjGBCL homologues are present in other echinoderm species, or other types of C-type lectins have cellular-aggregation-promoting activity.

In the experiment on autografting of coelomocytes into the eviscerated animal, a number of labeled coelomocytes were found to attach to the surface of the leftover digestive tract (**Figure 1**). It is not clear whether these cells moved and attached to the surface individually or, as in the *in vitro* observation, the cells formed aggregates and then moved to the surface. Some chemoattractants seem to be released from the surface of the digestive tract and/or the attached coelomocytes.

Gene expression analysis showed that *AjGBCL* is expressed in all the tissues that were examined. However, the aggregation-promoting effect was detected only in the extracts of the intestine and respiratory tree but not in those of other tissues. Because the gene expression analysis performed by us was not quantitative and the protein level was not proportionate to the mRNA level, the amount of lectin in tissues other than the intestine and respiratory tree, might be rather small. However, it cannot be concluded that the lectin does not contribute to wound sealing in these tissues. Coelomocytes are distributed not only in the coelom but also in the body wall and water vascular canals (32), and probably in other tissues as well. When tissues are damaged, only a small amount of lectin may be sufficient to induce coelomocytes proximate to the wounds to cause aggregation for the sealing of the wounds.

It was unexpected that the extracts of the Polian vesicle had an inhibitory effect on the aggregation. Polian vesicle is connected with a water vascular system (17); the inhibitory effect may contribute to suppress adverse aggregation of cells, which may result in clogging of the water vascular canal.

Interestingly, coelomocytes also express *AjGBCL* (**Figure 10**). The aggregation of coelomocytes was observed to begin immediately when the coelomic fluid was removed from the animal without any evident tissue damage. The present result suggests that the release of AjGBCL from coelomocytes triggers cellular aggregation in such a case as well. We are now confirming that the lectin exists in a particular type of cells, which may be the starter of aggregation in *A. japonicus*.

The aggregates formed in the presence of the intestinal extract were different not only in size but also in cellular composition compared to those in the control (**Figures 7**, **11**). When the extract was added, only a small number of free cells remained present around the aggregates in the presence of the intestinal piece (**Figure 2**), suggesting that most of the coelomocytes around the aggregates participated in aggregation. When the aggregates were dissociated by treatment with EDTA, the ratio of amoebocytes, which have been thought to be the major components of the aggregates in echinoderms, appeared to be lower than those in the control (**Figure 11**). In this condition, the ratio of two types of granulocytes was increased. This indicates that the chemoattractant signal released from the growing aggregates in the presence of the intestinal extract was quantitatively and/or qualitatively different.

In conclusion, we demonstrate that an endogenous factor, AjGBCL, promotes the aggregation of coelomocytes in *A. japonicus*. Future investigations will focus on AjGBCL-producing cells, receptors, and responses, including secretion of chemotactic agents, which will reveal molecular mechanisms underlying the aggregation of coelomocytes.

## Data Availability Statement

The raw sequencing data are available online in the DDBJ Sequence Read Archive (DRA accession number: DRA013169).

## Author Contributions

MT and ON conceived the project. CT performed RNA-seq and analyzed the data. MT performed all other experiments. MT wrote the original draft and ON and ST reviewed it. ON supervised the study. All authors contributed to the article and approved the submitted version.

## Conflict of Interest

The authors declare that the research was conducted in the absence of any commercial or financial relationships that could be construed as a potential conflict of interest.

## Publisher’s Note

All claims expressed in this article are solely those of the authors and do not necessarily represent those of their affiliated organizations, or those of the publisher, the editors and the reviewers. Any product that may be evaluated in this article, or claim that may be made by its manufacturer, is not guaranteed or endorsed by the publisher.

## Supplementary Material

Supplementary Video 1 Time-lapse observation of cellular aggregation in a piece of intestine placed on a glass slide. The piece of intestine was placed on a glass slide and the behavior of coelomocytes was observed. The piece is visible in the lower right of the visual field. Images were acquired every 10 min for a total of 1 h 30 min.
