# Supplementary material for: Galactose-Binding C-Type Lectin Promotes Cellular Aggregation of Coelomocytes in Sea Cucumber
Source: Front Immunol. 2021 Dec 14;12:783798. doi: 10.3389/fimmu.2021.783798 (PMC8713890; doi:10.3389/fimmu.2021.783798)
Supplement: Supplementary file 2 [file DataSheet_1.docx]

Galactose-binding C-type lectin promotes cellular aggregation of coelomocytes in sea cucumber

Mizuki Taguchi^1º^, Chikaya Tanaka^2^, Shigeyuki Tsutsui^1^, Osamu Nakamura^1*^

^1^School of Marine Biosciences, Kitasato University, Kanagawa, Japan

^2^Department of Biology, Tokyo Medical University, Tokyo, Japan

^º^ Present affiliation: Department of Biology, Research and Education Center for Natural Sciences, Keio University, Kanagawa, Japan

# Supplementary Figure


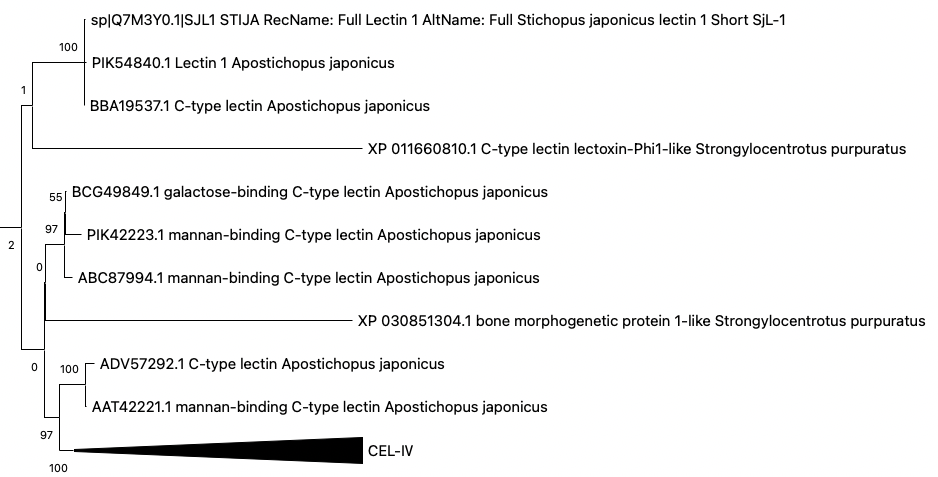


Supplementary Figure 1. **Phylogenetic analysis of AjGBCL**. Phylogenetic trees of C-type lectins of echinoderms using the maximum likelihood method. The percentage of trees in which associated taxa clustered together in the bootstrap test (100 replicates) is shown next to the branches.
